# Synthesis of *N*-arylpyridinium salts bearing a nitrone spin trap as potential mitochondria-targeted antioxidants

**DOI:** 10.1016/j.tet.2009.04.083

**Published:** 2009-07-04

**Authors:** Linsey Robertson, Richard C. Hartley

**Affiliations:** Centre for the Chemical Research of Ageing, WestCHEM Department of Chemistry, University of Glasgow, Glasgow G12 8QQ, UK

## Abstract

The generation of excess reactive oxygen species (ROS) in mitochondria is responsible for much of the oxidative stress associated with ageing (aging), and mitochondrial dysfunction is part of the pathology of neurodegeneration and type 2 diabetes. Lipophilic pyridinium ions are known to accumulate in mitochondria and this paper describes a general route for the preparation of nitrone-containing *N*-arylpyridinium salts having a range of lipophilicities, as potential therapeutic antioxidants. The compatibility of nitrones with the Zincke reaction is the key to their synthesis. Their trapping of carbon-centred radicals and the EPR spectra of the resulting nitroxides are reported.

## Graphical abstract

**Figure:**
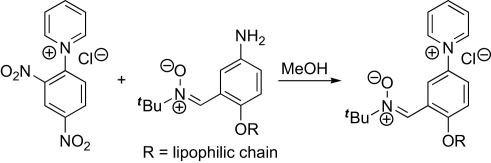

## 1 Introduction

**Scheme 1:**
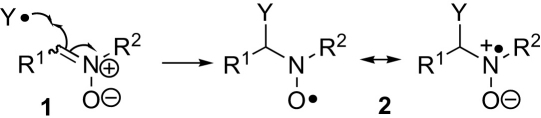
Spin trapping with nitrones.

The reactive oxygen species (ROS) generated within the mitochondria are ultimately responsible for much of the oxidative damage that leads to the neurodegeneration associated with ageing.^1^ ROS produced by dysfunctional mitochondria also contribute to increased risk of cardiovascular disease in people showing insulin resistance through type 2 diabetes, which is also associated with ageing.^2^ The life-expectancy of people is rising and the birth rate is low in most developed countries and this is leading to an ageing population so there is great interest in ensuring that people have a healthy old age. Nitrones have shown potential for the prevention and treatment of age-related diseases.^3^ One possible mechanism for their action is the scavenging of the free radicals that lead to oxidative damage. Nitrones **1** react with these highly reactive oxygen-centred and carbon-centred radicals (Y^•^) to give nitroxides **2** that are more stable and longer lived (Scheme 1).^4^ The nitroxides **2** can be detected by EPR spectroscopy and the hyperfine splittings observed can often be used to identify the radical that led to their formation. Thus, nitrones can be used as so-called spin traps for the study of radical processes in biological samples. Cyclic spin traps give observable nitroxides from both oxygen-centred and carbon-centred radicals. Acyclic spin traps such as *N*-*tert*-butyl-α-phenylnitrone, PBN (**1** R^1^=Ph, R^2^=*^t^*Bu), and *N*-*tert*-butyl-α-(*N*-oxypyrid-4-yl)nitrone, POBN (**1** R^1^=*N*-oxypyrid-4-yl, R^2^=*^t^*Bu), give long-lived adducts from carbon-centred radicals, and though the adducts of hydroxy and hydroperoxy radicals fragment rapidly, such traps are widely used with EPR spectroscopy^5^, ^6^ and continue to be developed for this purpose.^7^ Since acyclic nitrones have shown promise for the treatment of age-related diseases,^3^ they are also used as chemical interventions to study biological processes, and new acyclic nitrones continue to be developed as potential therapeutics.^8^ Stability to fragmentation would not be important to antioxidant activity if the overall pathway takes a reactive radical to benign products.

**Figure 1:**
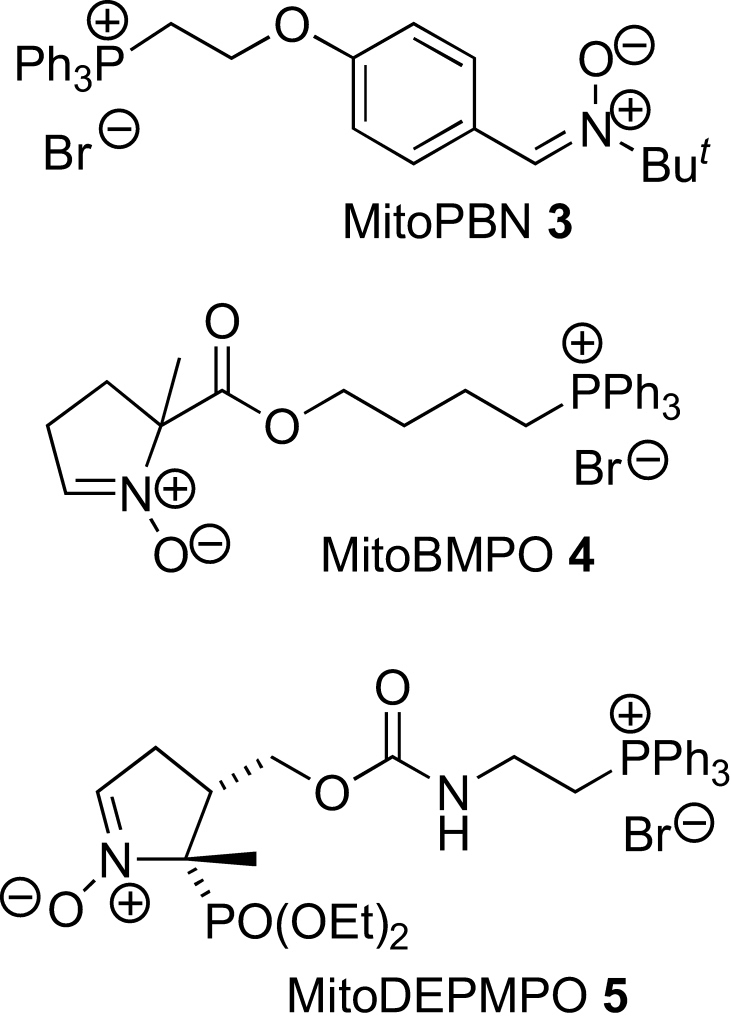
Mitochondria-targeted spin traps.

A few nitrone spin traps **3**–**5** have been designed to accumulate in mitochondria to scavenge and/or detect radicals generated there (Fig. 1).^9^ All bear the lipophilic alkyltriphenylphosphonium (TPP) cation, which has been pioneered as a targeting group for antioxidants by the groups of Murphy and Smith working in collaboration.^10^ TPP cations easily permeate biological membranes and accumulate up to a thousand-fold in the mitochondrial matrix due to the large mitochondrial membrane potential across the inner mitochondrial membrane set up by the electron transport chain. Although the TPP-group is effective and relatively non-toxic, it is not the only lipophilic cation that could act as a targeting group. We considered the pyridinium ion as a lower-molecular-weight alternative, and here report the synthesis of PBN-analogues that incorporate this group.

**Figure 2:**
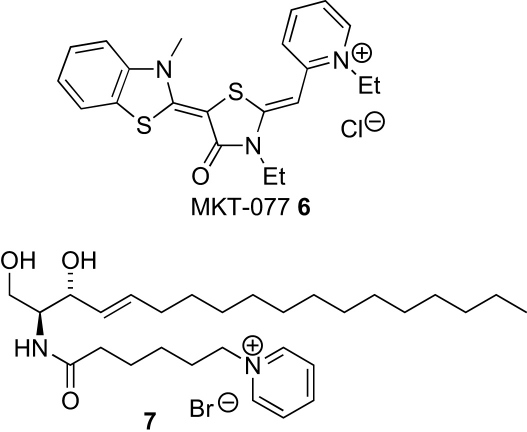
Pyridinium salts that accumulate in mitochondria.

There is precedent for the use of pyridinium ions in drug candidates to improve water solubility,^11^ but more importantly for us, pyridinium ions bearing lipophilic groups have been shown to accumulate in mitochondria.^12^, ^13^ These include the rhodacyanine dye MKT-077 **6**,^12^ which has anticarcinoma activity, and a ceramide derivative **7** that induces mitochondrial permeabilization (through the action of the ceramide moiety) (Fig. 2).^13^

**Figure 3:**
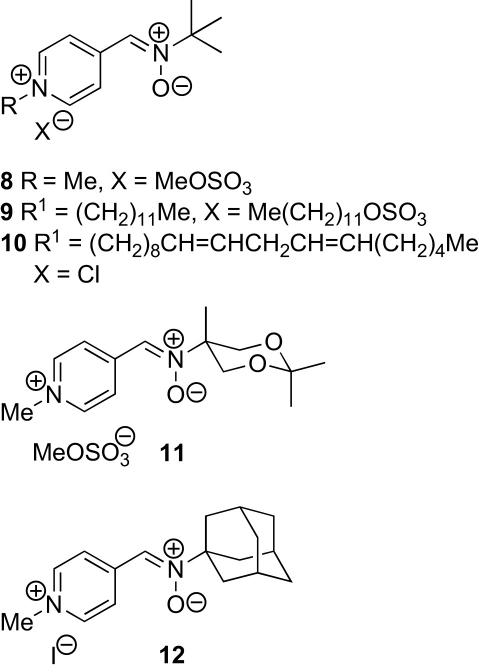
Pyridinium salts used as spin traps.

A few PBN-type nitrone spin traps derived from *N*-alkylpyridinium salts have been reported, but their use in targeting mitochondria has not been suggested. Janzen's team studied the behaviour of 2-, 3- and 4-(*N*-methylpyridinium) *N*-*tert*-butyl nitrones (2-MePyBN, 3-MePyBN and 4-MePyBN **8**, Fig. 3).^14^ These spin traps were water-soluble and stable for several days in aqueous solution and the spin adducts were marginally longer lived than those of PBN and 4-PyOBN at a range of pHs.^15^ The water-soluble^16^ 4-MePyBn **8** has since been used to study the chemical effects of ultrasound.^17^ The *N*-dodecyl derivative **9** was also prepared and was found to be almost insoluble in water,^14^ and the related lipophilic *N*-linoleyl derivative **10** was used by Hill and Thornalley as a membrane-bound spin trap for studying the generation of phenyl radicals when erythrocytes were treated with phenylhydrazine.^18^ Interestingly, the resulting spectrum was isotropic, showing that the nitroxide produced was not immobilised in the membrane.^19^ Nitrones **11** and **12** in which the *tert*-butyl group is replaced with a bulkier, more lipophilic substituent have also have also been reported.^14^, ^20^

Nitrones derived from pyridinium salts have also been used for purposes other than spin trapping. Some *N*-methylpyridinium nitrones have been tested for anticancer activity,^21^ and a nitronyl nitroxide pyridinium salt has been prepared as a spin-labelled version of the biological co-factor nicotinamide adenine dinucleotide (NAD^+^).^22^ Related pyridinium salts have been investigated as new materials, for example, in studies of molecule-based mangnetism, and nitronyl nitroxide radical units have been combined with an *N*-methylpyridinium ion to allow the ionic bonding necessary for molecular packing.^23^

We reasoned that nitrones derived from *N*-arylpyridinium salts would have a greater hydrophobic surface in the region of the cation than primary alkylpyridinium salts **8**–**12** and would be excellent candidates for mitochondria-targeted antioxidants. Placing the electron-withdrawing nitrone on a different aromatic ring from the pyridinium unit should reduce the propensity for biological reduction of the pyridinium ion relative to nitrones **8**–**12**.

**Figure 4:**
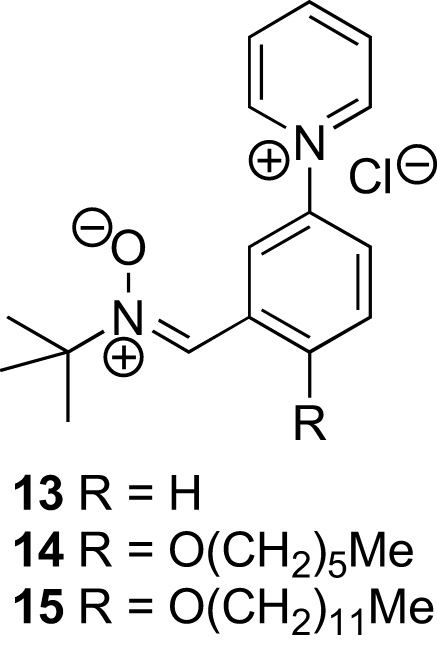

**Scheme 2:**
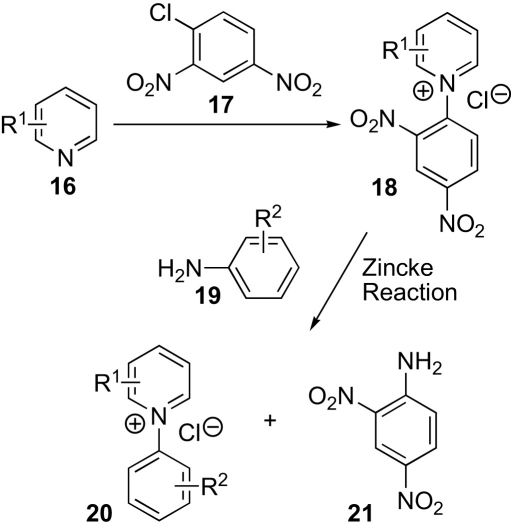
Preparation of pyridinium salts.

We decided to prepare three nitrones **13**–**15** that have increasing lipophilicity (Fig. 4). The key reaction in the synthesis of these *N*-arylpyridinium salts is the Zincke reaction,^24^, ^25^ which requires a two-step procedure (Scheme 2). First a pyridine **16** is reacted with 1-chloro-2,4-dinitrobenzene **17** to give an *N*-(2,4-dinitrophenyl)pyridinium chloride **18**, and this is then reacted with an aniline **19** to give a new *N*-arylpyridinium salt **20** and 2,4-dinitroaniline **21**. The mechanism of this second step, which is the Zincke reaction, involves nucleophilic attack by the nitrogen atom of the aniline at C-2 of the pyridinium ion, ring opening, *E*–*Z* interconversions, ring closure, and elimination of the 2,4-dinitroaniline **21**. In order for the formation of the *N*-(2,4-dinitrophenyl)pyridinium chloride **18** to proceed well, the pyridine must be nucleophilic, but if it is too electron-rich the Zincke reaction with the aniline **19** is impeded, particularly if the latter bears electron-withdrawing groups. Thus, the sequence will work best if R^2^ is electron-donating, but R^1^ must be neither too electron-withdrawing for the first step nor too electron-donating for the second.

**Scheme 3:**
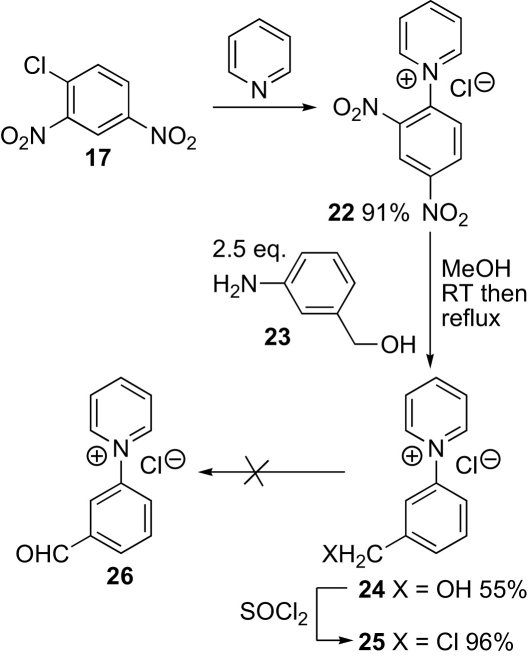
First approach.

The first target was nitrone **13**, which corresponds to the cationic head group alone. 1-Chloro-2,4-dinitrobenzene **17** reacted smoothly with pyridine to give pyridinium salt **22**. Zincke reaction with the electron-rich aniline **23** in methanol was straightforward, but the resulting benzylic alcohol **24** failed to oxidise to the aldehyde **26** under a variety of conditions. The oxidations were made difficult by the poor solubility of the alcohol in organic solvents other than methanol and DMSO together with the difficulty of separating different pyridinium salts from each other and the high water solubility of the pyridinium salts (adaptations of oxidation procedures to accommodate the solubility of the pyridinium salt **24** included using manganese dioxide,^26^ IBX (stabilised),^27^ hydrogen peroxide and iron(III) bromide,^28^ Swern and Parikh–Doering oxidation^29^). Conversion into the benzylic chloride **25** with neat thionyl chloride and heating with sodium bicarbonate in DMSO^30^ also failed to give the desired product **26** (Scheme 3).

**Scheme 4:**
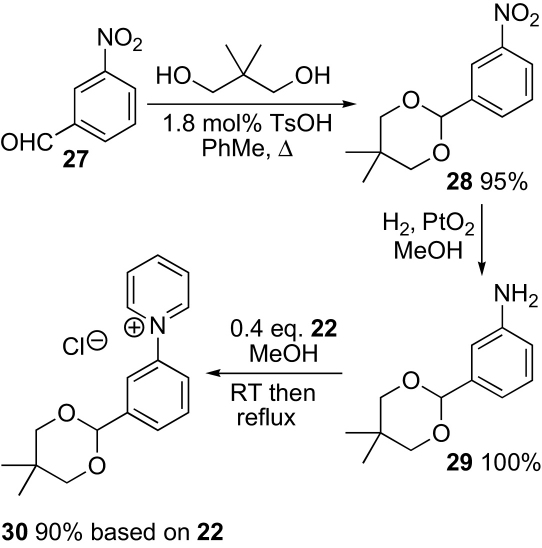
Second approach.

**Scheme 5:**
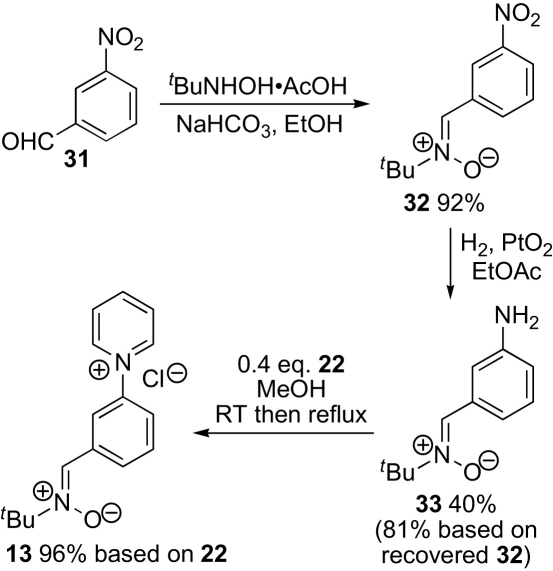
Successful route.

Next, introduction of the aldehyde masked as an acetal was attempted (Scheme 4). 3-Nitrobenzaldehyde **27** was converted into acetal **28**. Hydrogenation to give aniline **29** was carried out using platinum oxide to avoid the acidity associated with Pd–C. Aniline **29** underwent the Zincke reaction smoothly with the *N*-(2,4-dinitrophenyl)pyridinium salt **22** to give the pyridinium salt **30** in high yield. Unfortunately, removal of the acetal groups proved less straightforward with the 5,5-dimethyl-1,3-dioxane **30** failing to cleave in 6 M aqueous hydrochloric acid (conditions chosen for ease of isolating the water-soluble pyridinium salt product). Fortunately, at this stage an alternative route had been successful (Scheme 5). 3-Nitrobenzaldehyde **31** was converted into the *N*-*tert*-butylnitrone **32**. Selective hydrogenation of the nitro group then gave aniline **33**, which underwent the Zincke reaction with the *N*-(2,4-dinitrophenyl)pyridinium salt **22** to give the desired water-soluble nitrone **13**. Some reduction of the nitrone was observed if the hydrogenation of the nitro compound was driven to completion and the over-reduced side products were difficult to remove and led to paramagnetic contaminants in the final product **13**. Fortunately, the starting nitro compound **32** was easily crystallised from a mixture of nitrones **32** and **33** in ethanol–water when the hydrogenation was stopped after consuming about half the starting material, and this proved to be the best procedure.

**Scheme 6:**
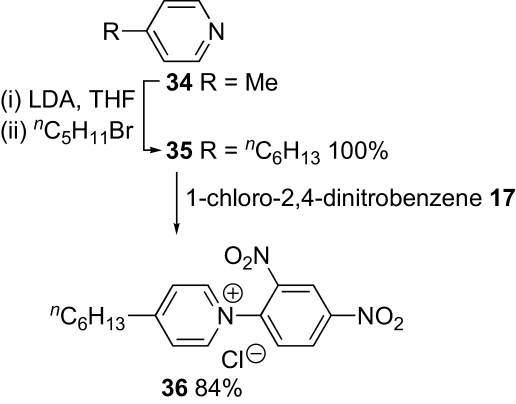

**Scheme 7:**
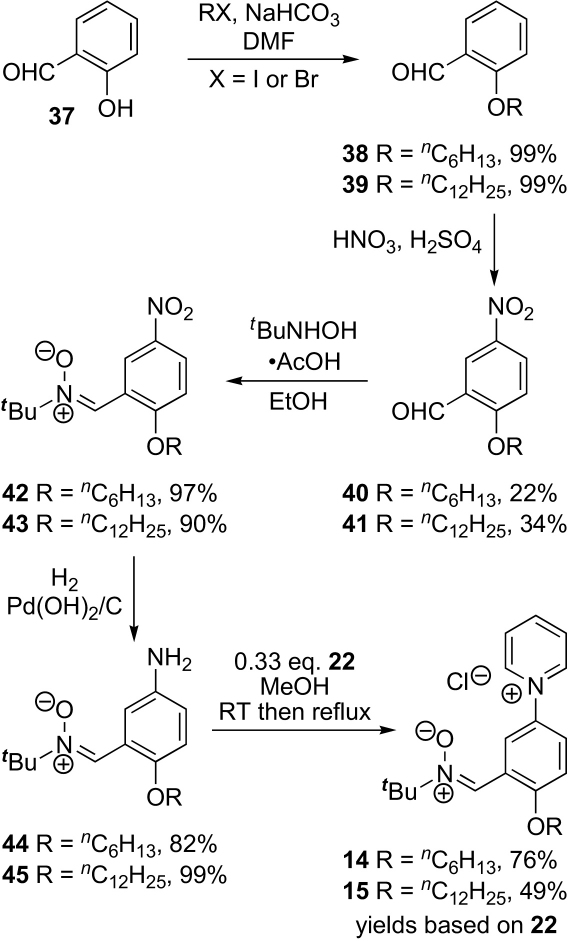

With a route to the *N*-arylpyridinium head group in hand, we set about modifying it to include a lipophilic tail. Initially 4-methylpyridine **34** was converted into the 4-hexyl derivative **35** by lithiation–alkylation (Scheme 6). This readily formed an *N*-(2,4-dinitrophenyl)pyridinium salt **36**, but the latter did not react with the aniline **33** bearing the electron-withdrawing nitrone group. Clearly, the combination of a less electron-rich pyridine and a more electron-rich aniline would be better. Therefore, salicylaldehyde **37** was converted into 2-hexyloxybenzaldehyde **38** and dodecyloxybenzaldehyde **39** in high yield. Nitration gave the corresponding nitro compounds **40** and **41** in modest yield after separation from other nitrated products. Conversion to nitrones **42** and **43** proceeded smoothly and hydrogenation, optimised through a change of catalyst, gave anilines **44** and **45**, respectively. Although aniline **44** was isolated as a 5:1 mixture with nitro compound **42**, this did not present a problem in the next step. Gratifyingly, the Zincke reactions between these anilines and the *N*-(2,4-dinitrophenyl)pyridinium salt **22** now gave the desired nitrones **14** and **15** (Scheme 7).

**Scheme 8:**
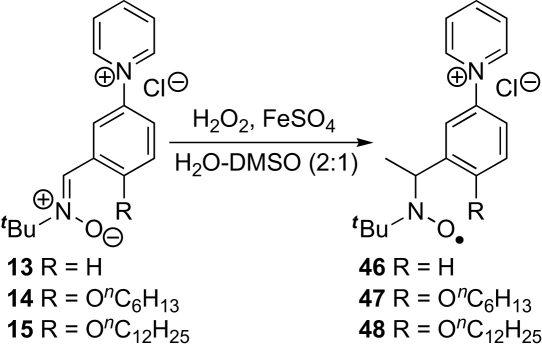

**Figure 5:**
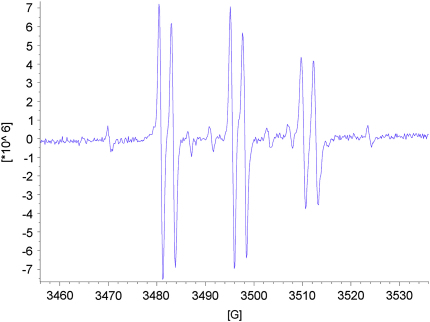
EPR spectrum of nitroxide **46***g*=2.0069, *A*_N_=14.70 G (t), *A*_H_^β^=2.45 G (d).

**Figure 6:**
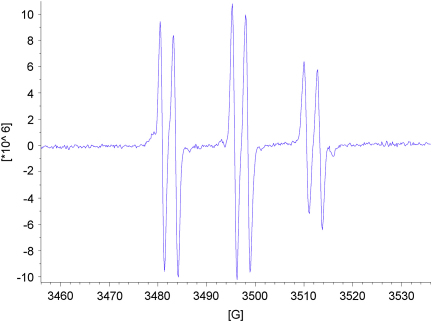
EPR spectrum of nitroxide **47***g*=2.0057, *A*_N_=14.83 G (t), *A*_H_^β^=2.70 G (d).

**Figure 7:**
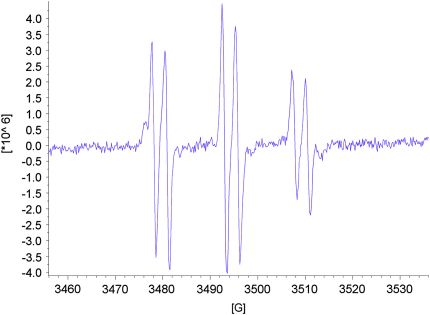
EPR spectrum of nitroxide **48***g*=2.0057, *A*_N_=14.90 G (t), *A*_H_^β^=2.75 G (d).

**Figure 8:**
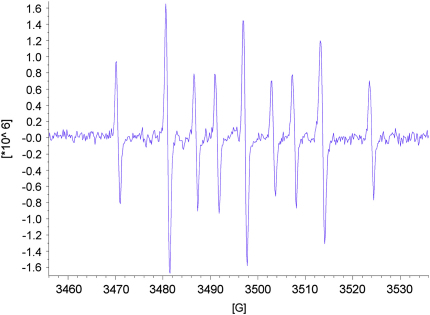
EPR spectrum of nitroxide **49***g*=2.0056, *A*_N_=16.32 G (t), *A*_H_^β^=10.35 G (t).

**Figure 9:**
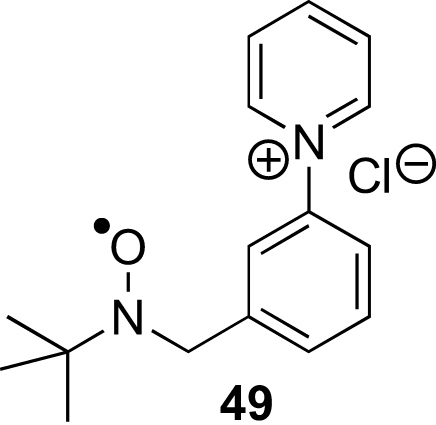
Proposed nitroxide contaminant.

As expected for acyclic nitrone spin traps,^4^ trapping of oxygen-centred radicals did not give stable nitroxides. On the other hand, when the nitrones **13**–**15** were reacted with methyl radicals generated from the reaction between DMSO and hydroxyl radicals produced under Fenton conditions from hydrogen peroxide and iron(II) sulfate (Scheme 8),^4^ EPR spectra were obtained that were consistent with nitroxides **46**–**48** (Figure 5, Figure 6, Figure 7). The high field lines are broadened due to *m*_I_-dependent linewidth effects and incomplete averaging of the anisotropic components caused by restricted tumbling of the spin adducts. Interestingly, the middle doublet shows the greatest peak height for nitroxides **47** and **48**, which contain the long aliphatic tails, whereas, for nitroxide **46**, the lowest field doublet is tallest. A weak background signal was observed when samples of nitrone **13** were dissolved in DMSO–water (Fig. 8), presumably due to nitroxide **49** (Fig. 9), which would result from some reduction of the nitrone moiety of aniline **33** to a hydroxylamine during the hydrogenation of nitro compound **32**, followed by air-oxidation to the nitroxide. Nitrone **13** produced under the optimised procedure was estimated to contain about 2% of this impurity by integration of the signal at *δ* 1.42 ppm in the ^1^H NMR spectrum, presumed to result from its *tert*-butyl group. The EPR signal for nitroxide **49** is visible in the EPR spectrum of nitroxide **46** (Fig. 5) because EPR spectroscopy is extremely sensitive and because the nitrone is used in excess (2.5 equiv with respect to the hydrogen peroxide) so that while the nitroxide **49** is already present, not all the nitrone **13** is converted into nitroxide **46**.

In conclusion, we have reported a new type of spin trap that bears an *N*-arylpyridinium ion, which should cause the more lipophilic members of the family **14** and **15** to accumulate in the matrix of mitochondria to act as antioxidants there. The nitrones **14** and **15** react with methyl radicals to give nitroxides **47** and **48** that give strong EPR signals, so they could potentially be used to detect carbon-centred radicals within mitochondria. The synthesis demonstrates the selective reduction of nitro groups in the presence of nitrones, the compatibility of nitrones with the Zincke reaction conditions, and illustrates the fine balance that has to be struck with regard to electron-donating and electron-withdrawing groups in this reaction.

## 2 Experimental

### 2.1 Synthesis

All reactions under an inert atmosphere were carried out using oven dried or flame dried glassware. Solutions were added via syringe. Diethyl ether, tetrahydrofuran, dichloromethane and toluene were dried where necessary using a solvent drying system, Puresolv™, in which solvent is pushed from its storage container under low nitrogen pressure through two stainless steel columns containing activated alumina and copper. Methanol was dried by distillation from magnesium and iodine, and then stored over 3 Å molecular sieves. Reagents were obtained from commercial suppliers and used without further purification unless otherwise stated. ^1^H and ^13^C NMR spectra were obtained on a Bruker DPX/400 spectrometer operating at 400 and 100 MHz, respectively. All coupling constants are measured in hertz. DEPT was used to assign the signals in the ^13^C NMR spectra as C, CH, CH_2_ or CH_3_. Mass spectra (MS) were recorded on a Jeol JMS700 (MStation) spectrometer. Infra-red (IR) spectra were obtained using attenuated total reflectance (ATR) so that the IR spectrum of the compound (solid or liquid) could be directly detected (thin layer) without any sample preparation.

### 2.2 *N*-*tert*-Butyl-α-[3-(pyrid-1′-yl)phenyl]nitrone chloride **13**

*N*-*tert*-Butyl-α-(3-aminophenyl)nitrone **33** (80 mg, 0.44 mmol) was added to a stirred solution of *N*-(2′,4′-dinitrophenyl)pyridinium chloride **22** (42 mg, 0.15 mmol) in anhydrous methanol (4 mL) under argon at rt. After 2 h the resulting red mixture was heated to reflux for 18 h until the red colour disappeared. The mixture was cooled, diluted with H_2_O and washed with EtOAc until no further colour was removed from the aqueous layer. The aqueous portion was concentrated in vacuo to give pyridinium salt **13** as a brown oil (40 mg, 96%). *δ*_H_ (400 MHz, MeOD): 1.68 (9H, s, 3×CH_3_), 7.87 (1H, t, *J* 8.0 Hz, H-5), 7.97 (1H, ddd, *J* 8.1, 2.4 and 0.9 Hz, H-6), 8.21 (1H, s, CH=N), 8.37 (2H, dd, *J* 7.9 and 6.9 Hz, H-3′ and H-5′), 8.49 (1H, dt, *J* 7.9 and 1.4 Hz, H-4), 8.87 (1H, tt, *J* 7.9 and 1.3 Hz, H-4′), 9.16 (1H, dd, *J* 2.0 and 1.8 Hz, H-2), 9.35 (2H, dd, *J* 6.9 and 1.4 Hz, H-2′ and H-6′). *δ*_C_ (100 MHz, MeOD): 28.37 (CH_3_), 73.17 (C), 125.14 (CH), 127.13 (CH), 129.67 (CH), 131.72 (CH), 132.00 (CH), 133.42 (CH), 134.57 (C), 144.38 (C), 146.12 (CH), 148.18 (CH). LRMS (FAB^+^) 255 [M^+^ (pyridinium cation), 100%]. HRMS: 255.1501, C_16_H_19_N_2_O requires 255.1497. *ν*_max_ (ATR) 3074 (CH), 2980 (CH), 2934 (CH), 1628 (Ar), 1583 (Ar), 1472 (Ar), 1190 (nitrone) cm^−1^.

### 2.3 *N*-*tert*-Butyl-α-[2-hexyloxy-5-(pyrid-1′-yl)phenyl]nitrone chloride **14**

α-(5-Amino-2-hexyloxyphenyl)-*N*-*tert*-butylnitrone **44** [556 mg of a mixture (5:1 mole ratio) of nitrones **44** and **42**, 1.56 mmol] was added to a stirred solution of *N*-(2′,4′-dinitrophenyl)pyridinium chloride **22** (180 mg, 0.64 mmol) in anhydrous methanol (14 mL) under argon at rt. After 2 h the resulting red mixture was heated to reflux for 18 h until the red colour disappeared. The mixture was cooled, diluted with H_2_O and washed with DCM until no further colour was removed from the aqueous layer. The aqueous portion was concentrated in vacuo to give **14** as an orange oil (189 mg, 76%). *δ*_H_ (400 MHz, MeOD): 0.93 (3H, t, *J* 6.8 Hz, CH_3_), 1.36–1.44 (4H, m, 2×CH_2_), 1.52–1.60 (11H, m, 3×CH_3_ and CH_2_), 1.87–1.94 (2H, m, CH_2_), 4.24 (2H, t, *J* 6.2 Hz, CH_2_), 7.40 (1H, d, *J* 9.0 Hz, H-3), 7.90 (1H, dd, *J* 9.0 and 3.0 Hz, H-4), 8.32 (1H, s, CH=N), 8.34 (2H, dd, *J* 7.9 and 6.8 Hz, H-3′ and H-5′), 8.76 (1H, tt, *J* 7.9 and 1.3 Hz, H-4′), 9.23 (2H, dd, *J* 6.9 and 1.3 Hz, H-2′ and H-6′), 9.62 (1H, d, *J* 3.0 Hz, H-6). *δ*_C_ (100 MHz, MeOD): 14.54 (CH_3_), 23.86 (CH_2_), 27.11 (CH_2_), 28.49 (CH_3_), 30.13 (CH_2_), 32.80 (CH_2_), 70.90 (CH_2_), 73.38 (C), 114.15 (CH), 122.28 (C), 124.92 (CH), 126.49 (CH), 128.91 (CH), 129.76 (CH), 136.89 (C), 146.03 (CH), 147.71 (CH), 160.45 (C). LRMS (FAB^+^) 355 [M^+^ (pyridinium cation), 100%]. HRMS: 355.2389, C_22_H_31_O_2_N_2_ requires 355.2386. *ν*_max_ (ATR) 2924 (CH), 2855 (CH), 1626 (aromatic), 1479 (aromatic), 1462 (aromatic), 1271 (nitrone), 1230 (C–O stretch) cm^−1^.

### 2.4 *N*-*tert*-Butyl-α-[2-dodecyloxy-5-(pyrid-1′-yl)phenyl]nitrone chloride **15**

α-(5-Amino-2-dodecyloxyphenyl)-*N*-*tert*-butylnitrone **45** (517 mg, 1.38 mmol) was added to a stirred solution of *N*-(2′,4′-dinitrophenyl)pyridinium chloride **22** (156 mg, 0.55 mmol) in anhydrous methanol (12 mL) under argon at rt. After 2 h the resulting red mixture was heated to reflux for 18 h until the red colour disappeared. The mixture was cooled, diluted with H_2_O and washed with DCM until no further colour was removed from the aqueous layer. The aqueous portion was concentrated in vacuo to give nitrone **15** as an orange oil (129 mg, 49%). *δ*_H_ (400 MHz, MeOD): 0.94 (3H, t, *J* 6.7 Hz, CH_3_), 1.34–1.67 (27H, m, 3×CH_3_+9×CH_2_), 1.93–2.00 (2H, m, CH_2_), 4.30 (2H, t, *J* 6.2 Hz, CH_2_), 7.46 (1H, d, *J* 9.0 Hz, H-3), 7.95 (1H, dd, *J* 9.0 and 3.0 Hz, H-4), 8.33 (2H, dd, *J* 7.8 and 6.8 Hz, H-3′ and H-5′), 8.34 (1H, s, CH=N), 8.82 (1H, tt, *J* 7.8 and 1.3 Hz, H-4′), 9.30 (2H, dd, *J* 6.9 and 1.3 Hz, H-2′ and H-6′), 9.69 (1H, d, *J* 3.0 Hz, H-6). *δ*_C_ (100 MHz, MeOD): 14.48 (CH_3_), 23.74 (CH_2_), 27.31 (CH_2_), 28.39 (CH_3_), 30.06 (CH_2_), 30.45 (CH_2_), 30.47 (CH_2_), 30.71 (CH_2_), 30.76 (CH_2_), 30.79 (CH_2_), 33.79 (CH_2_), 70.73 (CH_2_), 73.22 (C), 113.97 (CH), 122.21 (C), 124.76 (CH), 126.17 (CH), 128.70 (CH), 129.61 (CH), 136.76 (C), 145.91 (CH), 147.56 (CH), 160.28 (C). LRMS (FAB^+^) 439 [M^+^ (pyridinium cation), 100%]. HRMS: 439.3322, C_28_H_43_O_2_N_2_ requires 439.3325. *ν*_max_ (ATR) 2920 (CH), 2850 (CH), 1626 (aromatic), 1481 (aromatic), 1468 (aromatic), 1271 (nitrone), 1238 (C–O stretch) cm^−1^.

### 2.5 *N*-(2′,4′-Dinitrophenyl)pyridinium chloride **22**

Pyridine (4.0 mL, 49 mmol) and 1-chloro-2,4-dinitrobenzene (10.01 g, 49.4 mmol) were heated together at 95 °C for 1 h. The resulting yellow solid was triturated with acetone until no further colour was removed, to give pyridinium salt **22** as an off-white solid (12.6 g, 91%). Mp 193–195 °C. *δ*_H_ (400 MHz, MeOD): 8.39 (1H, d, *J* 8.7 Hz, H-6′), 8.47 (2H, dd, *J* 7.9 and 6.8 Hz, H-3 and H-5), 8.97 (1H, dd, *J* 8.7 and 2.5 Hz, H-5′), 9.02 (1H, tt, *J* 7.9 and 1.3 Hz, H-4), 9.31 (1H, d, *J* 2.5 Hz, H-3′), 9.40 (2H, dd, *J* 6.9 and 1.3 Hz, H-2 and H-6). *δ*_C_ (100 MHz, MeOD): 121.35 (CH), 128.01 (CH), 130.20 (CH), 132.01 (CH), 138.72 (C), 143.05 (C), 146.08 (CH), 148.79 (CH), 149.00 (C). LRMS (FAB^+^) 246 [M^+^ (pyridinium cation), 100%]. HRMS: 246.0514, C_11_H_8_N_3_O_4_ requires 246.0515. *ν*_max_ (KBr) 3117, 3057, 1610 (aromatic), 1542 (NO_2_), 1473 (aromatic), 1342 (NO_2_) cm^−1^. ^1^H NMR and ^13^C NMR data consistent with literature data obtained in (CD_3_)_2_SO.^31^

### 2.6 *N*-(3′-Hydroxymethylphenyl)pyridinium chloride **24**

3-Aminobenzyl alcohol **23** (2.74 g, 22.3 mmol) was added to a stirred solution of *N*-(2′,4′-dinitrophenyl)pyridinium chloride **22** (2.50 g, 8.90 mmol) in anhydrous methanol (60 mL) under argon at rt. After 24 h, the resulting red mixture was heated to reflux for 48 h until the red colour disappeared. The mixture was cooled, diluted with H_2_O and washed with EtOAc until no further colour was removed from the aqueous layer. The aqueous layer was concentrated in vacuo to give a brown solid that was recrystallised from *^i^*PrOH–acetone to give pyridinium salt **24** as brown cubes (1.08 g, 55%). Mp 107–109 °C. *δ*_H_ (400 MHz, MeOD): 4.83 (2H, s, C*H_2_*OH), 7.75–7.79 (3H, m, H-4′, H-5′ and H-6′), 7.85 (1H, br s, H-2′), 8.33 (2H, dd, *J* 7.8 and 6.8 Hz, H-3 and H-5), 8.84 (1H, tt, *J* 7.8 and 1.3 Hz, H-4), 9.30 (2H, dd, *J* 6.9 and 1.4 Hz, H-2 and H-6). *δ*_C_ (100 MHz, MeOD): 62.41 (CH_2_), 121.96 (CH), 122.54 (CH), 128.07 (CH), 129.14 (CH), 130.14 (CH), 143.08 (C), 144.59 (CH), 145.13 (C), 146.39 (CH). LRMS (FAB^+^) *186* [M^+^ (pyridinium cation), 100%]. HRMS: 186.0916, C_12_H_12_NO requires 186.0919. *ν*_max_ (ATR) 3295 (OH), 2916 (CH), 2851 (CH), 1614 (aromatic), 1471 (aromatic) cm^−1^.

### 2.7 *N*-(3′-Chloromethylphenyl)pyridinium chloride **25**

A mixture of *N*-(3′-hydroxymethylphenyl)pyridinium chloride **24** (300 mg, 1.61 mmol) and SOCl_2_ (3.0 mL, 40.6 mmol) was heated at 95 °C under argon for 18 h. The reaction was cooled and the excess SOCl_2_ was quenched by slow addition of H_2_O. The reaction mixture was washed with CHCl_3_ and the aqueous portion was concentrated in vacuo to give pyridinium salt **25** as a brown oil (310 mg, 96%). *δ*_H_ (400 MHz, MeOD): 4.88 (2H, s, CH_2_Cl), 7.79–7.89 (3H, m, H-4′, H-5′ and H-6′), 8.01 (1H, s, H-2′), 8.36 (2H, m, H-3 and H-5), 8.86 (1H, t, *J* 7.7 Hz, H-4), 9.32 (2H, d, *J* 6.1 Hz, H-2 and H-6). *δ*_C_ (100 MHz, MeOD): 45.49 (CH_2_), 125.42 (CH), 125.77 (CH), 129.66 (CH), 132.12 (CH), 132.85 (CH), 142.41 (CH), 144.53 (C), 146.12 (C), 148.10 (CH). LRMS (FAB^+^) 204 [M^+^ (^35^Cl, pyridinium cation), 100%], 206 [M^+^ (^37^Cl, pyridinium cation), 33%]. HRMS: 204.0579 and 206.0553. C_12_H_11_^35^ClN requires 204.0580 and C_12_H_11_^37^ClN requires 206.0553. *ν*_max_ (ATR) 3032 (CH), 2958 (CH), 1627 (aromatic), 1473 (aromatic) cm^−1^.

### 2.8 5,5-Dimethyl-2-(3′-nitrophenyl)-1,3-dioxane **28**

3-Nitrobenzaldehyde **27** (1.00 g, 6.62 mmol), 2,2-dimethyl-1,3-propanediol (1.41 g, 13.6 mmol) and *p*-toluenesulfonic acid (24 mg, 0.12 mmol) were dissolved in anhydrous toluene (23 mL). The reaction was heated under argon in a Dean–Stark apparatus at 140 °C for 24 h. Upon completion, the mixture was cooled, washed with NaHCO_3_ (×3), H_2_O and brine. The organic extracts were combined, dried (MgSO_4_), and concentrated in vacuo to give acetal **28** as a yellow oil (1.50 g, 95%) that solidified on standing. Mp 46–48 °C. *δ*_H_ (400 MHz, CDCl_3_): 0.81 (3H, s, CH_3_), 1.27 (3H, s, CH_3_), 3.67 (2H, d, *J* 10.6 Hz, CH_2_O–), 3.78 (2H, d, *J* 10.1 Hz, CH_2_O–), 5.45 (1H, s, CHO_2_), 7.53 (1H, t, *J* 8.0 Hz, H-5′), 7.81–7.83 (1H, m, H-4′), 8.18 (1H, ddd, *J* 1.0, 2.3 and 8.2 Hz, H-6′), 8.37 (1H, t, *J* 1.9 Hz, H-2′). *δ*_C_ (100 MHz, CDCl_3_): 21.86 (CH_3_), 23.07 (CH_3_), 30.31 (C), 77.70 (CH_2_), 99.92 (CH), 121.62 (CH), 123.69 (CH), 129.30 (CH), 132.49 (CH), 140.61 (C), 148.23 (C). LRMS (CI^+^) 238 [(M+H)^+^, 78%], 79 (100). HRMS: 238.1078, C_12_H_16_NO_4_ requires (M+H)^+^, 238.1079. *ν*_max_ (ATR) 2955 (CH), 2870 (CH), 1529 (NO_2_), 1460 (aromatic), 1348 (NO_2_), 1082 (C–O stretch) cm^−1^. ^1^H NMR and mp not in agreement with literature.^32^

### 2.9 2-(3′-Aminophenyl)-5,5-dimethyl-1,3-dioxane **29**

5,5-Dimethyl-2-(3′-nitro-phenyl)-[1,3]dioxane **28** (825 mg, 3.48 mmol) and platinum(IV) oxide (16 mg, 5 mol %) were dissolved in ethyl acetate (16.5 mL). The solution was flushed with hydrogen then placed under a hydrogen atmosphere and stirred at rt for 20 h. The catalyst was removed by filtration through cotton wool and the solution was concentrated in vacuo to give amine **29** as an orange-brown solid (720 mg, 100%). *δ*_H_ (400 MHz, CDCl_3_) 0.78 (3H, s, CH_3_), 1.30 (3H, s, CH_3_), 3.58–3.64 (4H, m, NH_2_+CH_2_), 3.74–3.77 (2H, m, CH_2_), 5.30 (1H, s, CHO_2_), 6.61 (1H, ddd, *J* 0.9, 2.4 and 7.9 Hz, H-6′), 6.83 (1H, t, *J* 2.0 Hz, H-2′), 6.86–6.89 (1H, m, H-4′), 7.13 (1H, t, *J* 7.8 Hz, H-5′). *δ*_C_ (100 MHz, MeOD): 21.84 (CH_3_), 23.06 (CH_3_), 30.19 (C), 77.58 (CH_2_), 101.78 (CH), 112.67 (CH), 115.61 (CH), 116.31 (CH), 129.18 (CH), 139.55 (C), 146.53 (C). LRMS (EI^+^) 207 (M^+^^•^, 90%), 121 (100). HRMS: 207.1263, C_12_H_17_NO_2_ requires 207.1259. *ν*_max_ (ATR) 3381 (NH_2_), 3360 (NH_2_), 2957 (CH), 1620 (NH_2_ bend), 1462 (aromatic), 1385, 1094 (C–O stretch) cm^−1^; mp 60–61 °C.

### 2.10 *N*-[3′-(5″,5″-Dimethyl-1″,3″-dioxan-2″-yl)phenyl]pyridinium chloride **30**

*N*-2,4-Dinitrophenyl pyridinium chloride **22** (333 mg, 1.18 mmol) was dissolved in anhydrous methanol (12 mL) and amine **29** (613 mg, 2.96 mmol) was added. The reaction was stirred under argon at rt for 20 h and then heated at reflux for 3 h until the red colour disappeared. The mixture was cooled, diluted with H_2_O and washed with EtOAc until no further colour was removed from the aqueous layer. The aqueous portion was concentrated in vacuo to give pyridinium salt **30** as a yellow oil (326 mg, 90%). *δ*_H_ (400 MHz, MeOD): 0.87 (3H, s, CH_3_), 1.30 (3H, s, CH_3_), 3.81 (4H, s, 2×CH_2_), 5.65 (1H, s, CHO_2_), 7.80 (1H, t, *J* 7.6 Hz, H-5′), 7.86 (1H, ddd, *J* 8.0, 2.4 and 1.3 Hz, H-4′ or H-6′), 7.91 (1H, dt, *J* 7.6 and 1.5 Hz, H-4′ or H-6′), 7.98–8.00 (1H, m, H-2′), 8.32 (2H, dd, *J* 7.9 and 6.9 Hz, H-3 and H-5), 8.83 (1H, tt, *J* 1.4 and 7.9 Hz, H-4), 9.29 (2H, dd, *J* 6.9 and 1.4 Hz, H-2 and H-6). *δ*_C_ (100 MHz, MeOD): 21.91 (CH_3_), 23.32 (CH_3_), 78.57 (CH_2_), 101.09 (CH), 123.45 (CH), 125.73 (CH), 129.53 (CH), 130.66 (CH), 131.58 (CH), 143.29 (C), 144.38 (C), 146.20 (CH), 147.97 (CH). LRMS (FAB^+^) 270 [M^+^ (pyridinium cation), 100%]. HRMS: 270.1488, C_17_H_20_NO_2_ requires 270.1494. *ν*_max_ (ATR) 3007 (CH), 2949 (CH), 2868 (CH), 1628 (aromatic), 1473 (aromatic) cm^−1^.

### 2.11 *N*-*tert*-Butyl-α-(3-nitrophenyl)nitrone **32**

3-Nitrobenzaldehyde **31** (500 mg, 3.31 mmol), *N*-(*tert*-butyl)hydroxylammonium acetate (740 mg, 4.96 mmol) and sodium hydrogen carbonate (417 mg, 4.96 mmol) were dissolved in ethanol (40 mL). The reaction was heated, with stirring, at 70 °C for 48 h. The reaction mixture was poured into H_2_O (120 mL) and left to stand for 1 h. The bright yellow crystals that formed were filtered off to give nitrone **32** as yellow, feathery crystals (641 mg, 92%). Mp 102–103 °C (lit.^33^ 108–110 °C). *δ*_H_ (400 MHz, CDCl_3_): 1.62 (9H, s, 3×CH_3_), 7.55 (1H, t, *J* 8.1 Hz, H-5), 7.69 (1H, s, CH=N), 8.18 (1H, dd, *J* 8.1 and 1.7 Hz, H-4), 8.57 (1H, dt, *J* 8.0 and 1.48 Hz, H-6), 9.17 (1H, t, *J* 1.7 Hz, H-2). *δ*_C_ (100 MHz, CDCl_3_): 28.31 (CH_3_), 71.99 (C), 123.18 (CH), 124.29 (CH), 127.94 (CH), 129.46 (CH), 132.53 (C), 133.99 (CH), 148.18 (C). LRMS (EI^+^) 222 (M^+^^•^, 10%), 84 (30%), 57 (C_4_H_9_^+^, 100%). HRMS: 222.1003, C_11_H_14_N_2_O_3_ requires 222.1004. *ν*_max_ (KBr) 2985 (CH), 2940 (CH), 1556 (aromatic), 1522 (NO_2_), 1415 (aromatic), 1366 (NO_2_), 1339 (nitrone) cm^−1^. ^1^H NMR data consistent with literature data obtained in (CD_3_)_2_SO.^33^

### 2.12 *N*-*tert*-Butyl-α-(3-aminophenyl)nitrone **33**

*N*-*tert*-Butyl-α-(3-nitrophenyl)nitrone **32** (500 mg, 2.34 mmol) and platinum(IV) oxide (11 mg, 5 mol %) were dissolved in ethyl acetate (10 mL). The solution was flushed with hydrogen then placed under a hydrogen atmosphere and stirred at rt for 45 min. The catalyst was removed by filtration through Celite and the solution was concentrated in vacuo to give a 1:1.4 mixture of the product **33** and starting material **32**. The starting material **32** was removed by precipitation from EtOH–H_2_O (×2) as a crystalline solid. The supernatant was concentrated to give a 10:1 mixture of nitrones **33** and **32** as a yellow oil (108 mg, 40%; 81% based on recovered SM). *δ*_H_ (400 MHz, CDCl_3_): 1.49 (9H, s, 3×CH_3_), 3.73 (2H, br s, NH_2_), 6.63 (1H, ddd, *J* 7.9, 2.4 and 0.9 Hz, H-4), 7.07 (1H, t, *J* 7.9 Hz, H-5), 7.19 (1H, d, *J* 7.8 Hz, H-6), 7.37 (1H, s, CH=N), 8.00 (1H, t, *J* 1.9 Hz, H-2). Material was carried on to next stage with no further purification or analysis due to potential instability.

### 2.13 4-Hexylpyridine **35**

LDA (3.6 mL of 2 M in THF–heptane–ethylbenzene, 7.2 mmol) was added dropwise over 10 min to a stirred solution of 4-picoline **34** (0.5 mL, 5.34 mmol) in anhydrous THF (5 mL) under argon at −78 °C. After stirring for a further 30 min at −78 °C, a solution of 1-bromopentane (0.44 mL, 3.6 mmol) in anhydrous THF (5 mL) was added dropwise over 5 min and the mixture allowed to warm to rt and stirred for 20 h. Saturated aqueous NH_4_Cl solution (10 mL) and H_2_O (10 mL) were added and the mixture was extracted with EtOAc (×2). The combined organic extracts were with H_2_O, dried (MgSO_4_) and concentrated in vacuo to give a yellow oil. The crude residue was chromatographed on SiO_2_ using EtOAc–hexane (1:9) as the eluent to give 4-hexylpyridine **35** as a yellow oil (579 mg, 100%). *R_f_*=[EtOAc–hexane (3:7)]: 0.23. *δ*_H_ (400 MHz, CDCl_3_): 0.87 (3H, t, *J* 6.8 Hz, CH_3_), 1.23–1.34 (6H, m, 3×CH_3_), 1.57–1.64 (2H, m, CH_2_), 2.58 (2H, t, *J* 7.6 Hz, CH_2_), 7.10 (2H, d, *J* 5.9 Hz, H-3 and H-5), 8.47 (2H, d, *J* 5.8 Hz, H-2 and H-6). *δ*_C_ (100 MHz, CDCl_3_): 14.17 (CH_3_), 22.65 (CH_2_), 28.95 (CH_2_), 30.37 (CH_2_), 31.70 (CH_2_), 35.37 (CH_2_), 124.08 (CH), 149.52 (CH), 152.12 (C). LRMS (EI^+^) 163 (M^+^^•^, 30%), 93 [M^+^^•^−CH_3_(CH_2_)_2_CH=CH_2_, 100]. HRMS: 163.1358, C_11_H_17_N requires 163.1361. *ν*_max_ (ATR) 2955 (CH), 2928 (CH), 2857 (CH), 1603 (aromatic), 1415 (aromatic) cm^−1^. ^1^H NMR data agree with literature.^34^

### 2.14 4-Hexyl-*N*-(2′,4′-dinitrophenyl)pyridinium chloride **36**

4-Hexylpyridine **35** (2.27 g, 13.9 mmol) and 1-chloro-2,4-dinitrobenzene (5.62 g, 27.8 mmol) were heated together at 95 °C for 48 h. The reaction was cooled, dissolved in H_2_O and washed with EtOAc until no further colour was removed from the aqueous layer. The aqueous portion was concentrated in vacuo to give **36** as a dark brown oil (4.29 g, 84%). *δ*_H_ (400 MHz, MeOD): 1.02 (3H, t, *J* 7.0 Hz, CH_3_), 1.44–1.60 (6H, m, 3×CH_2_), 1.94–2.01 (2H, m, CH_2_), 3.24 (2H, t, *J* 7.6 Hz, CH_2_), 8.36 (2H, d, *J* 6.4 Hz, H-3 and H-5), 8.44 (1H, d, *J* 8.7 Hz, H-6′), 8.98 (1H, dd, *J* 8.6 and 2.3 Hz, H-5′), 9.29 (1H, d, *J* 2.4 Hz, H-3′), 9.31 (2H, d, *J* 6.5 Hz, H-2 and H-6). *δ*_C_ (100 MHz, MeOD): 14.46 (CH_3_), 23.53 (CH_2_), 29.92 (CH_2_), 30.79 (CH_2_), 32.59 (CH_2_), 37.24 (CH_2_), 124.08 (CH), 129.12 (CH), 131.20 (CH), 132.92 (CH), 139.96 (C), 144.56 (C), 146.11 (CH), 150.82 (C), 169.12 (C). LRMS (FAB^+^) 330 [M^+^ (pyridinium cation), 100%]. HRMS: 330.1452, C_17_H_20_O_4_N_3_ requires 330.1454. *ν*_max_ (ATR) 2928 (CH), 2859 (CH), 1610 (aromatic), 1537 (NO_2_), 1462 (aromatic), 1342 (NO_2_) cm^−1^.

### 2.15 2-Hexyloxybenzaldehyde **38**

1-Bromohexane (4.7 mL, 0.034 mol) was added to a solution of salicylaldehyde (3.0 mL, 0.028 mol) and K_2_CO_3_ (4.647 g, 0.034 mol) in DMF (40 mL). The reaction was heated, with stirring, at 130 °C for 20 h. The reaction was cooled, filtered and diluted with H_2_O. The mixture was extracted with EtOAc (×3) and the combined organic extracts were washed with 1 M KOH. The organic extracts were dried (MgSO_4_) and concentrated in vacuo to give aldehyde **38** as a yellow oil (5.768 g, 99%). *δ*_H_ (400 MHz, CDCl_3_): 0.86–0.89 (3H, m, CH_3_), 1.27–1.33 (4H, m, 2×CH_2_), 1.41–1.47 (2H, m, CH_2_), 1.76–1.83 (2H, m, CH_2_), 4.01 (2H, t, *J* 6.4 Hz, CH_2_), 6.93 (1H, d, *J* 8.4 Hz, H-3), 6.94 (1H, t, *J* 7.5 Hz, H-5), 7.47 (1H, ddd, *J* 8.5, 7.4 and 1.8 Hz, H-4), 7.78 (1H, dd, *J* 7.7 and 1.8 Hz, H-6), 10.48 (1H, s, CHO). *δ*_C_ (100 MHz, CDCl_3_): 14.00 (CH_3_), 22.57 (CH_2_), 25.72 (CH_2_), 29.02 (CH_2_), 31.50 (CH_2_), 68.46 (CH_2_), 112.47 (CH), 120.38 (CH), 124.81 (C), 128.05 (CH), 135.92 (CH), 161.56 (C), 189.78 (CH). LRMS (EI^+^) 206 (M^+^^•^, 15%), 122 (30%), 85 (65%), 83 (100%). HRMS: 206.1309, C_13_H_18_O_2_ requires 206.1307. *ν*_max_ (ATR) 2955 (CH), 2859 (CH), 1688 (C=O), 1599 (aromatic), 1456 (aromatic), 1240 (C–O stretch) cm^−1^. Literature reports microanalysis only.^35^

### 2.16 2-Dodecyloxybenzaldehyde **39**

1-Iodododecane (8.38 mL, 0.034 mol) was added to a solution of salicylaldehyde (3.0 mL, 0.028 mol) and K_2_CO_3_ (4.647 g, 0.034 mol) in DMF (40 mL). The reaction was heated, with stirring, at 130 °C for 20 h. The reaction was cooled, filtered and diluted with H_2_O. The mixture was extracted with EtOAc (×3) and the combined organic extracts were washed with 1 M KOH. The organic extracts were dried (MgSO_4_) and concentrated in vacuo to give aldehyde **39** as a yellow solid, which melts upon handling (8.0295 g, 99%). *δ*_H_ (400 MHz, CDCl_3_): 0.87 (3H, t, *J* 6.2 Hz, CH_3_), 1.22–1.35 (16H, m, 8×CH_2_), 1.43–1.47 (2H, m, CH_2_), 1.79–1.86 (2H, m, CH_2_), 4.05 (2H, t, *J* 6.4 Hz, CH_2_), 6.94–6.99 (2H, m, H-4 and H-5), 7.48–7.52 (1H, m, H-3), 7.81 (1H, d, *J* 7.6 Hz, H-6), 10.50 (1H, s, CHO). *δ*_C_ (100 MHz, CDCl_3_): 14.19 (CH_3_), 22.77 (CH_2_), 26.13 (CH_2_), 29.16 (CH_2_), 29.43 (CH_2_), 29.54 (CH_2_), 29.64 (CH_2_), 29.67 (CH_2_), 29.71 (CH_2_), 29.73 (CH_2_), 31.99 (CH_2_), 68.57 (CH_2_), 112.54 (CH), 120.48 (CH), 124.94 (C), 128.20 (CH), 135.96 (CH), 161.64 (C), 189.90 (CH). LRMS (EI^+^) 290 (M^+^^•^, 60%), 122 (100). HRMS: 290.2244, C_19_H_30_O_2_ requires 290.2246. *ν*_max_ (ATR) 2922 (CH), 2853 (CH), 1688 (C=O), 1599 (aromatic), 1458 (aromatic), 1240 (C–O stretch) cm^−1^.

### 2.17 2-Hexyloxy-5-nitrobenzaldehyde **40**

A mixture of fuming nitric acid (100%, *d*=1.52, 7 mL) and concentrated sulfuric acid (18.1 M, 7 mL) was cooled, with stirring, to −10 °C. 2-Hexyloxybenzaldehyde **38** (5.55 g, 269 mmol) was added dropwise to the mixture. The reaction was allowed to warm to rt. After 1 h the reaction was poured onto ice. The precipitate formed was filtered, washed with H_2_O and dissolved in Et_2_O. The ether solution was washed with H_2_O, saturated aqueous NaHCO_3_ (×3) and again with H_2_O. The organic extracts were dried (MgSO_4_) and concentrated in vacuo to give a yellow solid. This solid was recrystallised three times from Et_2_O–hexane to give aldehyde **40** as off-white needles (1.49 g, 22%). Mp 61–62 °C (lit.^36^ 66–70 °C). *δ*_H_ (400 MHz, CDCl_3_): 0.91 (3H, t, *J* 6.8 Hz, CH_3_), 1.35–1.38 (4H, m, 2×CH_2_), 1.47–1.52 (2H, m, CH_2_), 1.87–1.94 (2H, m, CH_2_), 4.21 (2H, t, *J* 6.4 Hz, CH_2_), 7.10 (1H, d, *J* 9.2 Hz, H-3), 8.41 (1H, dd, *J* 9.2 and 2.8 Hz, H-4), 8.69 (1H, d, *J* 2.8 Hz, H-6), 10.47 (1H, s, CHO). *δ*_C_ (100 MHz, CDCl_3_): 14.11 (CH_3_), 22.65 (CH_2_), 25.71 (CH_2_), 28.90 (CH_2_), 31.52 (CH_2_), 70.00 (CH_2_), 113.00 (CH), 124.64 (CH), 124.66 (C), 130.78 (CH), 141.49 (C), 165.39 (C), 187.77 (CH). LRMS (EI^+^) 251 (M^+^^•^, 85%), 84 (65), 43 (100). HRMS: 251.1157, C_13_H_17_NO_4_ requires 251.1158. *ν*_max_ (ATR) 2951 (CH), 2911 (CH), 2843 (CH), 1688 (C=O), 1609 (aromatic), 1512 (NO_2_), 1427 (aromatic), 1339 (NO_2_), 1273 (C–O stretch) cm^−1^.

### 2.18 2-Dodecyloxy-5-nitrobenzaldehyde **41**

A solution of 2-dodecyloxybenzaldehyde **39** (3.79 g, 13 mmol) in concentrated sulfuric acid (3 mL) was cooled, with stirring, to −10 °C. A mixture of 70% nitric acid (1.3 mL) and concentrated sulfuric acid (18.1 M, 1.3 mL) was also cooled, with stirring, to −10 °C. The acid mixture was added dropwise to the aldehyde solution and the mixture stirred at rt for 15 min. The reaction mixture was poured onto ice. The aqueous layers were extracted with EtOAc (×3). The combined organic extracts were washed with H_2_O (×3), dried (MgSO_4_) and concentrated in vacuo to give an orange oil. Chromatography on SiO_2_ using 5% EtOAc–hexane (1:19) as the eluent gave **41** as a yellow oil (1.141 g, 34%). *R_f_*=0.40 [EtOAc–hexane (1:4)]. *δ*_H_ (400 MHz, CDCl_3_): 0.83 (3H, t, *J* 6.5 Hz, CH_3_), 1.22–1.35 (16H, m, 8×CH_2_), 1.44–1.51 (2H, m, CH_2_), 1.85–1.92 (2H, m, CH_2_), 4.20 (2H, t, *J* 6.5 Hz, CH_2_), 7.10 (1H, d, *J* 9.2 Hz, H-3), 8.36 (1H, dd, *J* 9.2 and 2.8 Hz, H-4), 8.60 (1H, d, *J* 2.8 Hz, H-6), 10.42 (1H, s, CHO). *δ*_C_ (100 MHz, CDCl_3_): 14.14 (CH_3_), 22.72 (CH_2_), 25.94 (CH_2_), 28.86 (CH_2_), 29.30 (CH_2_), 29.38 (CH_2_), 29.54 (CH_2_), 29.59 (CH_2_), 29.66 (CH_2_), 31.62 (CH_2_), 31.94 (CH_2_), 69.99 (CH_2_), 113.04 (CH), 124.31 (CH), 124.54 (C), 130.64 (CH), 141.32 (C), 165.35 (C), 187.58 (CH). LRMS (EI^+^) 335 (M^+^^•^, 22%), 318 (45), 97 (49), 85 (70), 71 (88), 57 (C_4_H_9_^+^, 100). HRMS: 335.2096, C_19_H_29_NO_4_ requires 335.2097. *ν*_max_ (ATR) 2924 (CH), 2853 (CH), 1692 (C=O), 1609 (aromatic), 1589 (aromatic), 1522 (NO_2_), 1466 (aromatic), 1341 (NO_2_), 1271 (C–O stretch) cm^−1^.

### 2.19 *N*-*tert*-Butyl-α-(2-hexyloxy-5-nitrophenyl)nitrone **42**

2-Hexyloxy-5-nitrobenzaldehyde **40** (490 mg, 1.95 mmol), *N*-(*tert*-butyl)hydroxylammonium acetate (437 mg, 2.93 mmol) and sodium hydrogen carbonate (246 mg, 2.93 mmol) were dissolved in ethanol (20 mL). The reaction was heated, with stirring, at 70 °C for 72 h. The reaction mixture was poured into H_2_O (100 mL) and left to stand for 1 h. The resulting precipitate was filtered and dissolved in EtOAc. The solution was washed with H_2_O (×2) and brine, dried (MgSO_4_) and concentrated in vacuo to give a yellow solid. The solid was recrystallised from Et_2_O–hexane to give nitrone **42** as yellow cubes (610 mg, 97%). Mp 102–103 °C. *δ*_H_ (400 MHz, CDCl_3_): 0.92 (3H, t, *J* 6.7 Hz, CH_3_), 1.34–1.39 (4H, m, 2×CH_2_), 1.46–1.54 (2H, m, CH_2_), 1.63 (9H, s, 3×CH_3_), 1.85–1.92 (2H, m, CH_2_), 4.14 (2H, t, *J* 6.4 Hz, CH_2_), 6.95 (1H, d, *J* 9.2 Hz, H-3), 8.16 (1H, s, CH=N), 8.27 (1H, dd, *J* 9.1 and 2.6 Hz, H-4), 10.24 (1H, d, *J* 2.6 Hz, H-6). *δ*_C_ (100 MHz, CDCl_3_): 14.12 (CH_3_), 22.73 (CH_2_), 25.85 (CH_2_), 28.36 (CH_3_), 28.98 (CH_2_), 31.55 (CH_2_), 69.48 (CH_2_), 72.04 (C), 110.32 (CH), 120.54 (C), 123.33 (CH), 124.05 (CH), 126.87 (CH), 141.25 (C), 160.95 (C). LRMS (EI^+^) 322 (M^+^^•^, 20%), 266 (M^+^^•^−C_4_H_8_, 70), 57 (C_4_H_9_^+^, 100). HRMS: 322.1896, C_17_H_26_N_2_O_4_ requires 322.1893. *ν*_max_ (ATR) 2951 (CH), 2982 (CH), 1609 (aromatic), 1580 (aromatic), 1516 (NO_2_), 1466 (aromatic), 1343 (NO_2_), 1273 (nitrone), 1248 (C–O stretch) cm^−1^.

### 2.20 *N*-*tert*-Butyl-α-(2-dodecyloxy-5-nitrophenyl)nitrone **43**

2-Dodecyloxy-5-nitrobenzaldehyde **41** (1.41 g, 4.40 mmol), *N*-(*tert*-butyl)hydroxylammonium acetate (984 mg, 6.60 mmol) and sodium hydrogen carbonate (554 mg, 6.60 mmol) were dissolved in ethanol (40 mL). The reaction was heated, with stirring, at 70 °C for 48 h. The reaction mixture cooled, diluted with H_2_O and extracted with EtOAc (×3). The combined organic extracts were washed with H_2_O (×2) and brine (×1), dried (MgSO_4_) and concentrated in vacuo to give nitrone **43** as a yellow solid (1.60 g, 90%). Mp 39–40 °C. *δ*_H_ (400 MHz, CDCl_3_): 0.88 (3H, t, *J* 6.4 Hz, CH_3_), 1.27–1.40 (16H, m, 8×CH_2_), 1.47–1.52 (2H, m, CH_2_), 1.63 (9H, s, 3×CH_3_), 1.85–1.92 (2H, m, CH_2_), 4.13 (2H, t, *J* 6.4 Hz, CH_2_), 6.93 (1H, d, *J* 9.2 Hz, H-3), 8.09 (1H, s, CH=N), 8.24 (1H, dd, *J* 9.1 and 2.7 Hz, H-4), 10.28 (1H, d, *J* 2.7 Hz, H-6). *δ*_C_ (100 MHz, CDCl_3_): 14.25 (CH_3_), 22.81 (CH_2_), 26.18 (CH_2_), 28.39 (CH_3_), 29.03 (CH_2_), 29.40 (CH_2_), 29.46 (CH_2_), 29.68 (CH_2_), 29.73 (CH_2_), 29.75 (CH_2_), 29.77 (CH_2_), 32.03 (CH_2_), 69.45 (CH_2_), 72.01 (C), 110.27 (CH), 120.72 (C), 122.70 (CH), 123.92 (CH), 126.69 (CH), 141.28 (C), 160.87 (C). LRMS (EI^+^) 406 (M^+^^•^, 12%), 350 (70), 318 (65), 182 (73), 57 (C_4_H_9_^+^, 100). HRMS: 406.2829, C_23_H_38_N_2_O_4_ requires 406.2832. *ν*_max_ (ATR) 2955 (CH), 2920 (CH), 2851 (CH), 1607 (aromatic), 1518 (NO_2_), 1464 (aromatic), 1339 (NO_2_), 1271 (nitrone), 1244 (C–O stretch) cm^−1^.

### 2.21 α-(5-Amino-2-hexyloxyphenyl)-*N*-*tert*-butylnitrone **44**

*N*-*tert*-Butyl-α-(2-hexyloxy-5-nitrophenyl)nitrone **42** (613 mg, 1.90 mmol) and palladium hydroxide (20% on carbon, 66 mg, 5 mol %) were dissolved in ethyl acetate (9.5 mL). The solution was flushed with hydrogen then placed under a hydrogen atmosphere and stirred at rt for 30 min. The catalyst was removed by filtration through Celite and the solution was concentrated in vacuo to give a 5:1 mixture of α-(5-amino-2-hexyloxyphenyl)-*N*-*tert*-butylnitrone **44** and nitrone **42** as a yellow oil (556 mg, approx. 82% yield of nitrone **44**). Data derived for nitrone **44**: *δ*_H_ (400 MHz, CDCl_3_): 0.87 (3H, t, *J* 7.1 Hz, CH_3_), 1.29–1.34 (4H, m, 2×CH_2_), 1.40–1.47 (2H, m, CH_2_), 1.56 (9H, s, 3×CH_3_), 1.70–1.77 (2H, m, CH_2_), 3.45 (2 h, br s, NH_2_), 3.88 (2H, t, *J* 6.4 Hz, CH_2_), 6.64–6.69 (2H, m, H-5 and H-6), 8.01 (1H, s, CH=N), 8.78 (1H, d, *J* 2.6 Hz, H-2)]. Material was carried on to next stage with no further purification or analysis.

### 2.22 α-(5-Amino-2-dodecyloxyphenyl)-*N*-*tert*-butylnitrone **45**

*N*-*tert*-Butyl-α-(2-dodecyloxy-5-nitrophenyl)nitrone **43** (563 mg, 1.38 mmol) and palladium hydroxide (20% on carbon, 48 mg, 5 mol %) were dissolved in ethyl acetate (7.5 mL). The solution was flushed with hydrogen then placed under a hydrogen atmosphere and stirred at room temperature for 50 min. The catalyst was removed by filtration and the solution was concentrated in vacuo to give **45** as a brown solid (517 mg, 99%). *δ*_H_ (400 MHz, CDCl_3_): 0.87 (3H, t, *J* 6.3 Hz, CH_3_), 1.21–1.34 (16H, m, 8×CH_2_), 1.42–1.47 (2H, m, CH_2_), 1.59 (9H, s, 3×CH_3_), 1.73–1.78 (2H, m, CH_2_), 3.40 (2H, br s, NH_2_), 3.91 (2H, t, *J* 6.4 Hz, CH_2_), 6.69–6.73 (2H, m, H-5 and H-6), 8.05 (1H, s, CH=N), 8.82 (1H, d, *J* 2.3 Hz, H-2). The material was carried onto next stage with no further purification or analysis.

### 2.23 EPR spectroscopy

Iron(II) sulfate (100 μL of a 1 mM aqueous solution) and hydrogen peroxide (100 μL of a 1 mM aqueous solution) were added to a solution of the nitrone **13**, **14** or **15** in DMSO (100 μL of a 2.5 mM solution in the case of nitrone **13** and a 10 mM solution for nitrones **14** and **15**). The solution [0.83 mM nitrone **13** or 3.33 mM nitrone **14** or **15**, 0.33 mM hydrogen peroxide, 0.33 mM iron(II) sulfate in water–DMSO (2:1)] was then immediately transferred to a quartz flat cell and placed in the EPR spectrometer for analysis. Spectra were acquired on a Bruker e-scan™ bench-top EPR machine with a permanent magnet and a magnetic sweep circuit (centre of field=0.345 T, sweep width 25 mT) operating at a frequency of 9.8 GHz (X-band). Acquisition parameters: RG 3.99×10^3^, 2.76 mW, MA 0.5 G. Hyperfine couplings were derived from simulations using WINEPR SimFonia™.
